# Age effects on tinnitus and hearing loss in CBA/CaJ mice following sound exposure

**DOI:** 10.1186/2193-1801-3-542

**Published:** 2014-09-20

**Authors:** Ryan J Longenecker, Kurt T Chonko, Steve M Maricich, Alexander V Galazyuk

**Affiliations:** Department of Anatomy and Neurobiology, Northeast Ohio Medical University, 4209 State Route 44, Rootstown, OH 44272 USA; Kent State University, Kent, OH 44240 USA; Department of Biology, Case Western Reserve University, Cleveland, OH 44106 USA; Department of Pediatrics, Richard King Mellon Foundation Institute for Pediatric Research, University of Pittsburgh, Pittsburgh, PA 15224 USA

**Keywords:** Acoustic trauma, Gap-induced suppression of the acoustic startle reflex, Tinnitus development, Age-related hearing loss

## Abstract

Tinnitus is a maladaptive neuropathic condition that develops in humans and laboratory animals following auditory insult. In our previous study we demonstrated that sound exposure leads to development of behavioral evidence of tinnitus in a sample of exposed mice. However, this tinnitus mouse model did not account for long-term maladaptive plasticity or aging, factors that are commonly linked to the human tinnitus population. Therefore the same group of mice was monitored for tinnitus for 360 days post exposure. Tinnitus was assessed behaviorally by measuring gap-induced pre-pulse suppression of the acoustic startle (GPIAS). Cochlear histology was performed on both control (unexposed) and experimental mice to determine whether sound exposure caused any evident cochlear damage. We found that 360 days after exposure the vast majority of exposed mice exhibited similar gap detection deficits as detected at 84 days post exposure. These mice did not demonstrate significant loss of inner/outer hair cells or spiral ganglion neurons compared to the control sample. Lastly, we demonstrated that GPIAS deficits observed in exposed animals were unlikely exclusively caused by cochlear damage, but could be a result of central auditory maladaptive plasticity. We conclude that CBA/CaJ mice can be considered a good animal model to study the possible contribution of age effects on tinnitus development following auditory insult.

## Background

Tinnitus, a brain disorder that causes the perception of sound without an acoustic stimulus, afflicts more than 30% of Americans (Erlandsson et al. 1992; Ahmad and Seidman 2004). The prevalence of tinnitus has been shown to increase with age (Shargorodsky et al. 2010). Hearing loss is often a result of occupational or recreational noise exposure and is the strongest predictor of tinnitus development (König et al. 2006). Patients with tinnitus often fail to seek medical attention for their condition until many years after being exposed to noise (Rosenhall and Karlsson 1991). This delay could be explained by the time necessary for changes to be developed in the cochlea (Kujawa and Liberman 2009) or central auditory system (Munguia et al. 2013) following auditory insults. An animal model of chronic tinnitus would provide an invaluable tool for working towards understanding the complex neural mechanisms underlining its development.

The majority of tinnitus studies on animals have been completed between 28 days and 84 days after sound exposure (Middleton et al. 2011; Chen et al. 2013; Koehler and Shore 2013). While many of the basic elements of tinnitus have been elucidated in these studies, they don’t correlate well to the long term development and stabilization of tinnitus in human patients. Here, we investigate tinnitus in mice up to 360 days post exposure. This model approximates the stereotypical human condition by mimicking an exposure early in life with consequential development of tinnitus at an older age.

A limitation of many tinnitus studies is the requirement for operant conditioning to assess tinnitus, which is both labor and time intensive (Jastreboff and Sasaki 1994; Bauer and Brozoski 2001; Heffner and Harrington 2002; Lobarinas et al. 2004). Alternatively, a method that makes use of innate reflexes in response to an acoustic stimulus has provided researchers with a quick and effective way to assess tinnitus. The gap-induced prepulse inhibition of the acoustic startle (GPIAS) methodology has been used in rats (Turner and Parrish 2008; Kraus et al. 2011) and mice (Longenecker and Galazyuk 2011; Turner et al. 2012). These studies have provided valuable information regarding tinnitus development. In our previous research, we characterized the development of tinnitus over an 84 day time course after sound exposure in mice (Longenecker and Galazyuk 2011). We found that in the initial stages of tinnitus development, GPIAS deficits are widespread across frequencies. However, as tinnitus progressed from the initial stages to later stages, the GPIAS deficits started to center around a narrower frequency range (20-31 kHz), stabilizing 84 days post exposure. This behavioral evidence of tinnitus development is in agreement with electrophysiological markers of tinnitus (Mulders and Robertson 2011; Llano et al. 2012). Thus, it seems that tinnitus development in the first 84 days is a dynamic process. In the present study, we tracked each mouse from the original study for 360 days after sound exposure.

Animal models have elucidated the effects of age-related and noise-induced hearing loss that are highly prevalent in humans (Kujawa and Liberman 2006; Sha et al. 2008; Ohlemiller et al. 2010; Bao and Ohlemiller 2010; Turner et al. 2012). However, the effects of such deficits introduce potential confounds for the assessment of tinnitus in animals. The GPIAS method for tinnitus assessment is subject to key assumptions; that the animal being tested can react to a loud startling stimulus and that they can hear a the background noise that will inhibit the startle reflex. Critics of the GPIAS method have suggested that deficits in gap detection can be attributed to hearing loss at those background frequencies in which the gap is presented. It has been shown that acoustic trauma directly causes some degree of permanent hearing damage (Bohne and Clark 1982; Ou et al. 2000; Kujawa and Liberman 2009). However, not all patients with hearing loss develop tinnitus (Lockwood et al. 2002). While it is assumed that all animals have some degree of hearing deficits in response to acoustic trauma, a smaller percentage will develop tinnitus (Middleton et al. 2011; Longenecker and Galazyuk 2012; Singer et al. 2013; Chen et al. 2013; Koehler and Shore 2013). Interestingly, it is possible for humans to develop tinnitus without detectable hearing loss (Weisz et al. 2006). However, even in tinnitus patients that present clinically “normal” audiograms (Job et al. 2007), it would inappropriate to assume no damage exists either peripherally or centrally. These facts lead us to question whether the GPIAS method is sensitive enough to detect tinnitus despite an underlying hearing deficit. This research demonstrated that the mouse tinnitus model is reliable and furthermore permits detection of long term changes in gap detection performance.

## Results

**Figure 1:**
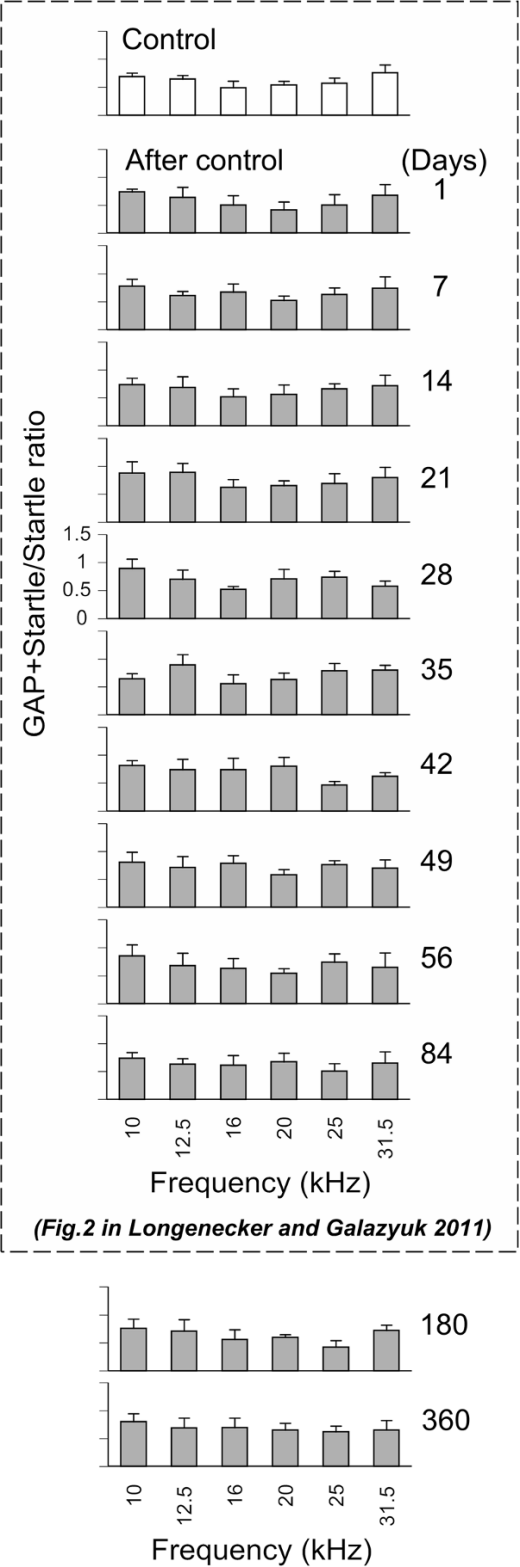
**Fluctuations of gap-induced suppression of the acoustic startle response over a 360 day period in a control mouse.** Open bars represent control means ± SD of (G + S)/S ratios measured at 6 different background frequencies (10, 12.5, 16, 20, 25, and 31.5 kHz). The grey bars represent ratios measured at the same frequencies but at different time points after pre-exposure measurements.

**Figure 2:**
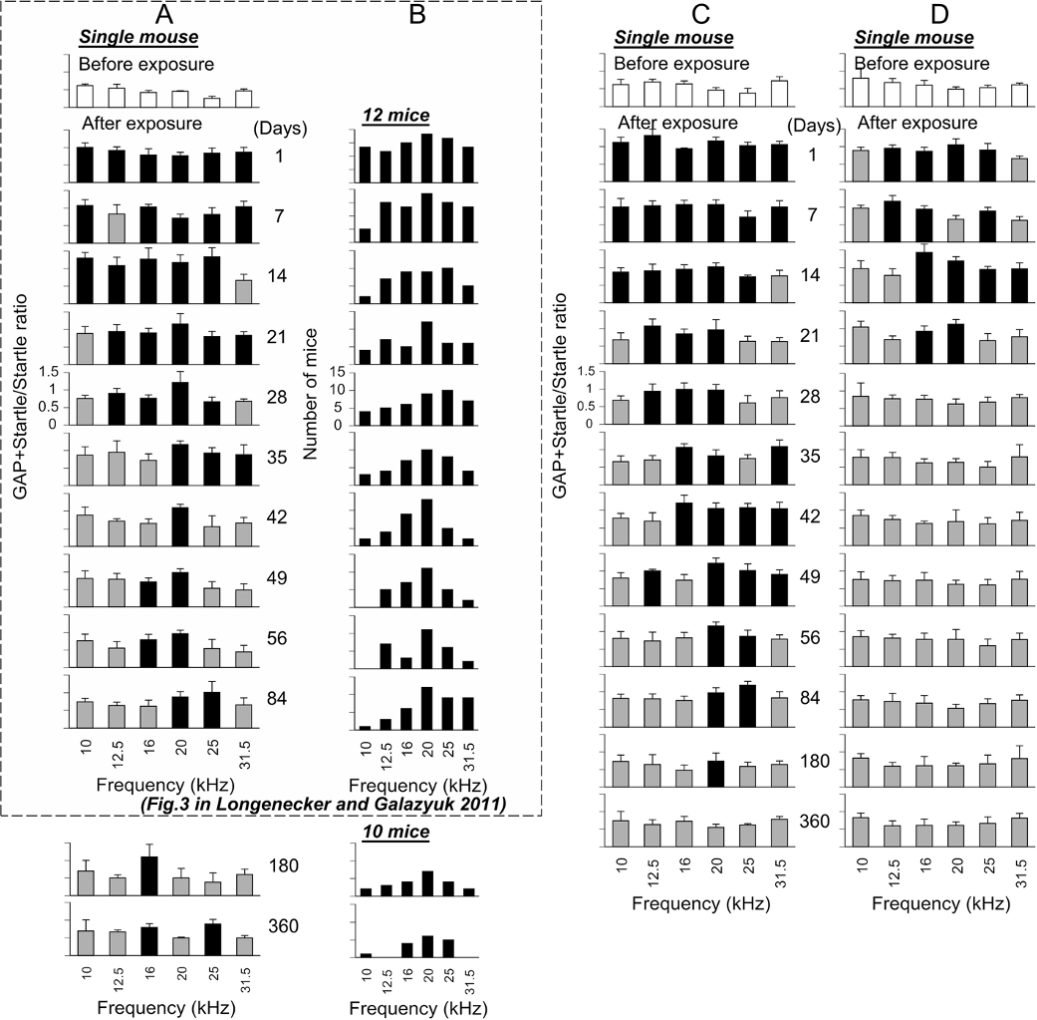
**Time-dependent changes of gap-induced suppression of the acoustic startle response in mice for a 360 day duration after sound exposure. (A)** Changes in gap detection performance in a single sound exposed mouse. Open bars represent mean ± SD of (G + S)/S ratios measured before sound exposure. Grey and black bars represent the ratios which were (black bars) or were not (grey bars) significantly different from the control. **(B)** The histogram depicts only significant increases in the ratios as a function of background frequency (e.g. indicated by black bars in A) obtained from a sample of 12 sound-exposed mice. 2 out of 12 mice were lost after the 84 day time point so only 10 mice are represented at 180 and 360 day time points. **(C)** Changes in gap detection performance in a mouse which performance was recovered to the pre exposed level between 180 and 360 days after exposure. **(D)** Changes in gap detection performance in a mouse which did not show gap detection deficits at 84 days post exposure and continued show no deficits up to 360 days.

**Figure 3:**
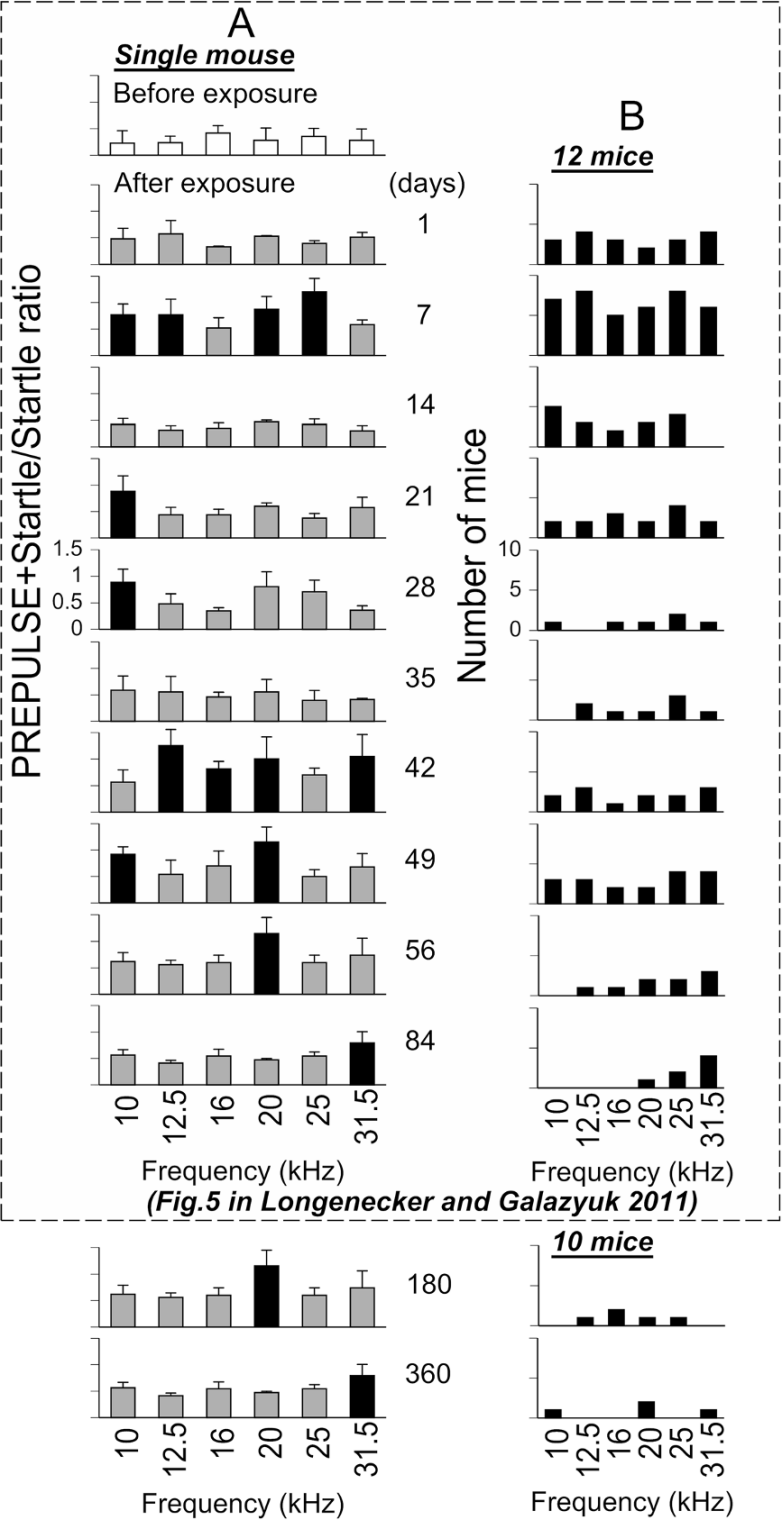
**Time-dependent changes of prepulse induced suppression of the acoustic startle response in mice for a 360 day duration after sound exposure. (A)** Changes of prepulse detection performance in a single mouse. Open bars represent mean ± SD of ratios measured before sound exposure. Grey and black bars represent the ratios which were (black bars) or were not (grey bars) significantly different from the control. **(B)** The histogram depicts only significant increases in the ratios as a function of background frequency obtained from a sample of 12 sound-exposed mice.

**Figure 4:**
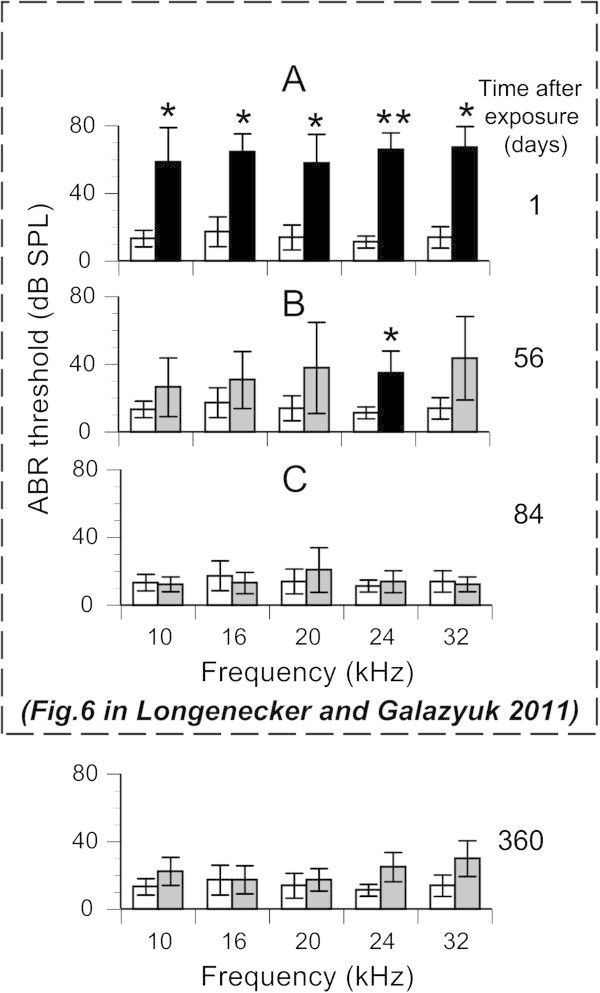
**Effects of sound exposure on ABR thresholds for 12 experimental mice. (A)** Mean ABR thresholds at five frequencies (10, 16, 20, 24, and 32 kHz) recorded before (open bars) and immediately after sound exposure (black bars). **(B & C)** Mean ABR thresholds recorded at 56, 84, and 360 days after exposure, respectively. Black and grey bars were and were not, respectively, significantly different from pre-sound exposure responses. *p < 0.05, **p < 0.001.

**Figure 5:**
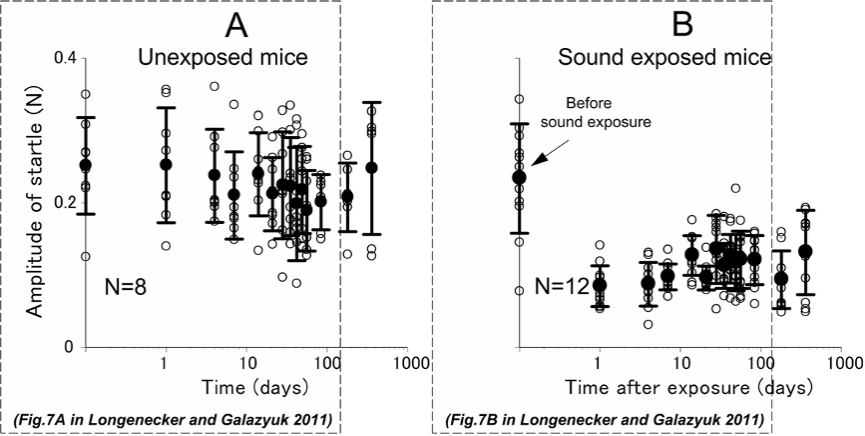
**Reduction in acoustic startle response amplitude after sound exposure. (A)** Individual data points (open circles) and mean ± SD values from 8 control mice recorded over a 360 day period. **(B)** The same data collected from 12 experimental mice before and at different times after sound exposure.

In our previous study we described the development of the behavioral evidence of tinnitus for an 84 day period following sound exposure (Longenecker and Galazyuk 2011). We found that tinnitus development was a highly dynamic process. Immediately after sound exposure, all animals showed gap detection deficits over a wide range of sound frequencies. However, about 56 days later, gap detection deficits were evident at a narrow frequency range (20–31 kHz). At that time point, it was not clear whether the development of tinnitus was completed, representing a chronic phase, or if it was still in the process of further development. To address this question, we continued monitoring the tinnitus development in the same group of mice for 360 days following sound exposure. Control mice were also monitored within the same time period. To demonstrate the development of tinnitus over a 360 day period in the same group of mice, Figures 1, 2, 3, 4 and 5 include some figures from Longenecker and Galazyuk 2011 showing data up to the 84 day time point.

### GAP detection performance in the control mice

Since we have observed the same group of mice for 360 days we wanted to make sure that these mice did not show any age-related decline in their gap detection performance within this time period. To test this hypothesis we measured and compared (G + S)/S ratios in the control group of mice at different time points within one year testing period. Each mouse in the control group (8) exhibited a robust gap-induced suppression of their startle response. Their (G + S)/S ratio values were averaged over all six background frequencies and ranged from 0.39 to 0.82 (mean ± SD = 0.66 ± 0.1). A representative mouse in Figure 1 showed similar (G + S)/S ratios for six different frequencies at 180 and 360 days which ranged from 0.42 to 0.76 (mean ± SD = 0.63 ± 0.12) and from 0.62 to 0.81 (mean ± SD = 0.68 ± 0.06), respectively. These ratios are not significantly different (p = 0.37). Furthermore, the ratio at the 360 day time point was not significantly different (p = 0.24) from the ratio recorded in our previous study at 84 days (Longenecker and Galazyuk 2011). The gap detection performance in each control mouse did not change significantly during 360 days of monitoring.

### GAP detection performance in the sound exposed mice

In our previous study we found that during the 84 days following sound exposure a vast majority of exposed mice (86%, 12/14) developed behavioral signs of tinnitus (Longenecker and Galazyuk 2011). The gap detection deficits were predominantly found between 20 and 31.5 kHz. These deficits suggest that these mice showed behavioral evidence of tinnitus at these frequencies. In the current study, we monitored the gap detection performance in these mice 360 days post exposure. We found that the vast majority of mice exhibiting behavioral signs of tinnitus at the 84 day mark (83%, 10/12) continued to demonstrate these signs at 180 and 360 days following sound exposure. A representative mouse in Figure 2 presented evidence of tinnitus at the 84 day mark (84 days) between 20 and 25 kHz. At the 180 day time point the range of deficits had shifted to 16 kHz. By 360 days, behavioral signs of tinnitus were evident in this mouse at 16 and 25 kHz. Figure 2B demonstrates the distribution of ratios that had significantly increased as a function of background frequency (e.g., indicated by black bars in Figure 2A), which were obtained from a sample of 10 out of 12 sound-exposed mice that showed signs of tinnitus up to 360 days of monitoring. The range of background frequencies where behavioral signs of tinnitus were present at 180 days was similar to those at 84 days. By 360 days following exposure, behavioral signs of tinnitus were predominantly evident within a narrow frequency range from 16 to 25 kHz.

Interestingly, 2 mice demonstrated an alternative pattern of gap detection deficits as they aged. Although they demonstrated signs of tinnitus at 84 day and 180 day time points, no behavioral deficits representing tinnitus were present when they were tested 360 days after sound exposure (Figure 2C). These mice showed a robust gap detection performance at all frequencies tested which was not significantly different from the performance before sound exposure.

It is important to note that 2 mice out of 14 did not develop behavioral signs of tinnitus within an 84 day period after sound exposure, and had no signs of tinnitus at 180 or 360 days after exposure (Figure 2D).

### Prepulse detection performance in the sound exposed mice

Prepulse inhibition of the acoustic startle reflex was assessed in control and sound-exposed mice at 180 or 360 days following sound exposure. The prepulse amplitude and narrow-band center frequencies were the same as the background noise used in the gap detection test.

Prepulse detection was used as a tool to determine whether gap detection deficits can be simply explained by hearing loss or by the presence of tinnitus. At 180 or 360 day time periods all mice in the control group exhibited robust prepulse inhibition at all frequencies tested. The average of ((P + S)/S) ratio values across all tested frequencies at 180 or 360 days were 0.64 and 0.62, respectively. Although not as robust as in the control mice, the sound exposed mice also demonstrated strong prepulse inhibition. A representative mouse in Figure 3A had an average ratio over all six background frequencies which varied from 0.38 to 1.1 (mean ± SD = 0.63 ± 0.25) at 180 days, and from 0.51 to 0.93 (mean ± SD = 0.68 ± 0.16) at 360 days after exposure. Gap detection results at 360 days were not significantly different from the results found at 84 days following sound exposure in our previous study. The sample data from 12 mice showed a similar trend of changes (Figure 3B). While some mice demonstrated prepulse detection deficits at 84 days after exposure (predominantly at high frequencies), these deficits became less pronounced at 180 days and even more so at 360 days following exposure (Figure 3B).

To further validate whether gap detection deficits observed in exposed mice can be explained by hearing loss, their PPI and GPIAS performances (shown in Figures 2B and 3B) were compared using a *t*-test. This comparison showed that at 5 out of 6 frequencies tested PPI and GPIAS performances were significant different (p < 0.04), whereas only at 10 kHz was this difference not significant (p = 0.062). The slightly higher P-value at 10 kHz can be explained by a relatively small sample size of PPI and GPIAS measurements which were compared for this frequency.

### Effects of sound exposure on ABR

ABR thresholds were measured in sound exposed mice 360 days following sound exposure at five different frequencies (10, 16, 20, 24, and 32 kHz). The ABR thresholds for all five frequencies were slightly elevated but not significantly different from the ABR thresholds recorded at 84 days following exposure (p = 0.061) or from the control measurements performed before sound exposure (p = 0.057).

### Effects of sound exposure on the acoustic startle response

In our previous study, we demonstrated that the magnitude of the startle response was suppressed after sound exposure, and remained suppressed up to 84 days following exposure (Longenecker and Galazyuk 2011). In the present study the amplitude of startle responses was measured for both the control and sound exposed mice at 180 or 360 days post-exposure. The startle response amplitude recorded in the control group at these time points varied among animals from 0.125 to 0.326 (mean ± SD = 0.248 ± 0.091) (Figure 5A). These amplitudes did not significantly differ from those previously collected during the first 84 days of testing (F(2,15) = 0.97, p = 0.402). Similarly, the range of startle amplitudes at 180 or 360 days were not significantly different (F(2,29) = 2.76, p = 0.08) from the range recorded during the first 84 days in exposed mice (Figure 5B).

### Effects of sound exposure on hair cells and spiral ganglion neurons

**Figure 6:**
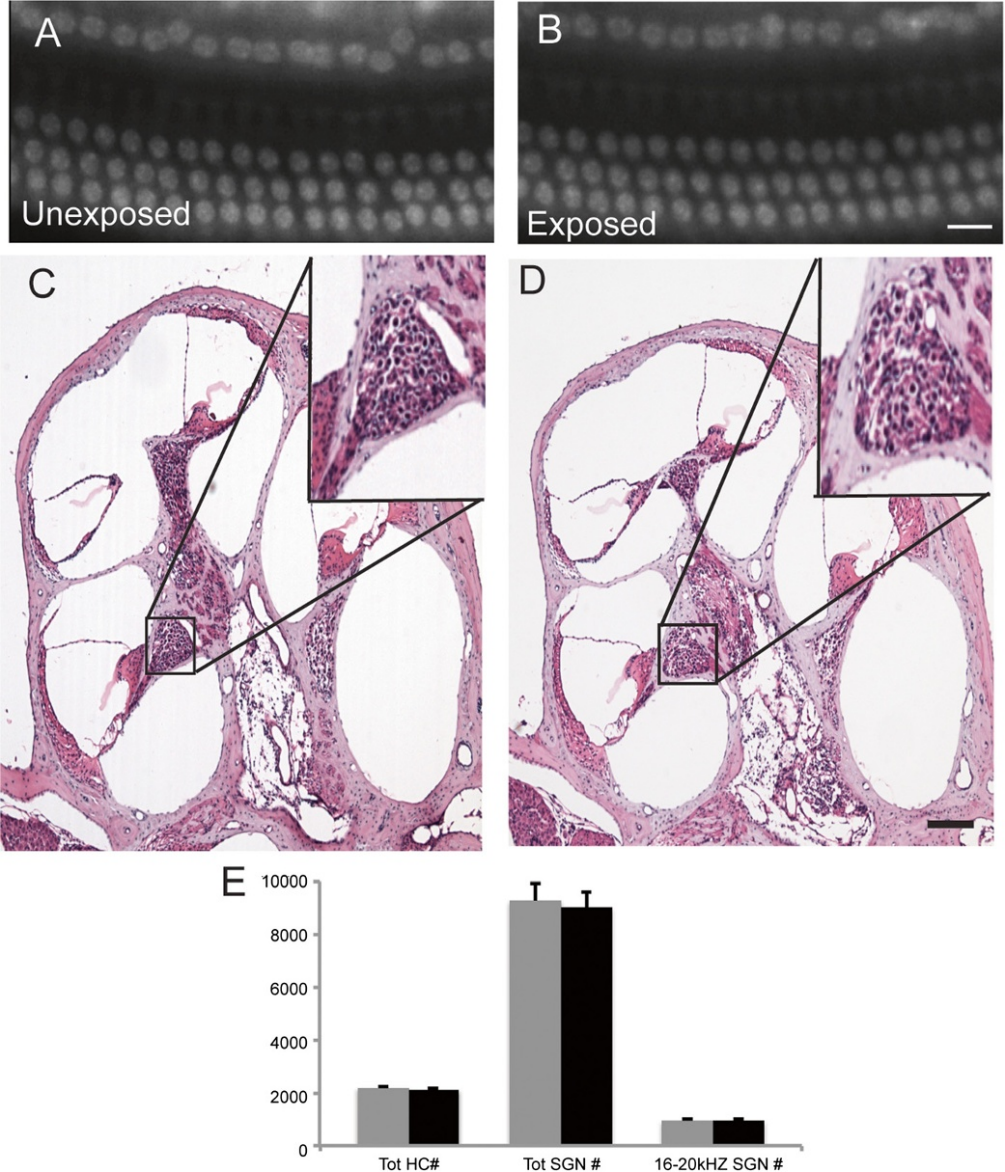
**Sound exposure did not change the number of hair cells or spiral ganglion neurons in the cochlea.** DAPI stained nuclei of hair cells in whole mount preparations of exposed **(A)** and unexposed **(B)** ears show no differences. H&E stained, mid-modiolar cochlear sections show no difference in spiral ganglion neurons of exposed **(C)** and unexposed **(D)** ears. **(E)** Cell counts for unexposed (gray) and exposed (black) ears. Scale bars: 15 μm (A, B, insets C, D)**;** 200 μm (C, D).

To determine whether acoustic trauma led to either hair cell or spiral ganglion neuron (SGN) loss, we counted the hair cells (n = 3 mice) and SGNs (n = 4 mice) in the exposed and unexposed ears. Total numbers of hair cells (2824 ± 44 vs. 2921 ± 50; p = 0.22), inner hair cells (703 ± 12 vs. 725 ± 9; p = 0.20), and outer hair cells (2120 ± 52 vs. 2196 ± 41; p = 0.32) were not significantly different in exposed vs. unexposed ears (n = 3 mice) (Figure 6). Similarly, the total numbers of SGNs (9011 ± 621 vs. 9279 ± 685; p = 0.78) and SGNs in the 16–20 kHz region (932 ± 75 vs. 925 ± 83; p = 0.95) were not significantly different between exposed and unexposed ears (Figure 6). We conclude that the acoustic trauma paradigm that we used in this study did not cause the loss of either of these cell populations.

### Potential effects of hearing loss caused by sound exposure on tinnitus assessment

Sound exposure for tinnitus induction, as well as using gap-induced inhibition of the acoustic startle reflex for tinnitus assessment is becoming a popular technique among scientists using an animal model for tinnitus research. However, potential hearing loss during sound exposure raises some doubts about the gap-induced inhibition of the acoustic startle reflex as a reliable method for tinnitus assessment. To address this issue we conducted a series of experiments on 4 sound exposed mice with and without behavioral evidence of tinnitus.

In agreement with other labs’ tinnitus animal model, our animals were exposed unilaterally. During exposure one ear was sound protected with a cotton plug, which is known to offer some protection during sound exposure (Turner et al. 2006). Such attenuation provides assurance that the level of sound exposure used for tinnitus induction (116 dB SPL during one hour) was unlikely to cause severe hearing loss in the protected ear. The goal of this study was to determine the feasibility that hearing loss caused by sound exposure in the exposed ear was responsible for the same gap detection deficits that are believed to be an indicator of tinnitus. Our assumption was that the tinnitus percept has a central rather than peripheral origin. If so, then the gap detection deficits associated with tinnitus should be present in either of the following conditions: When both ears are tested, or when only the protected ear is tested. To test the unexposed ear, the exposed ear was temporarily plugged during behavioral testing. Then, the gap detection performances in these two experimental conditions were analyzed and compared.

**Figure 7:**
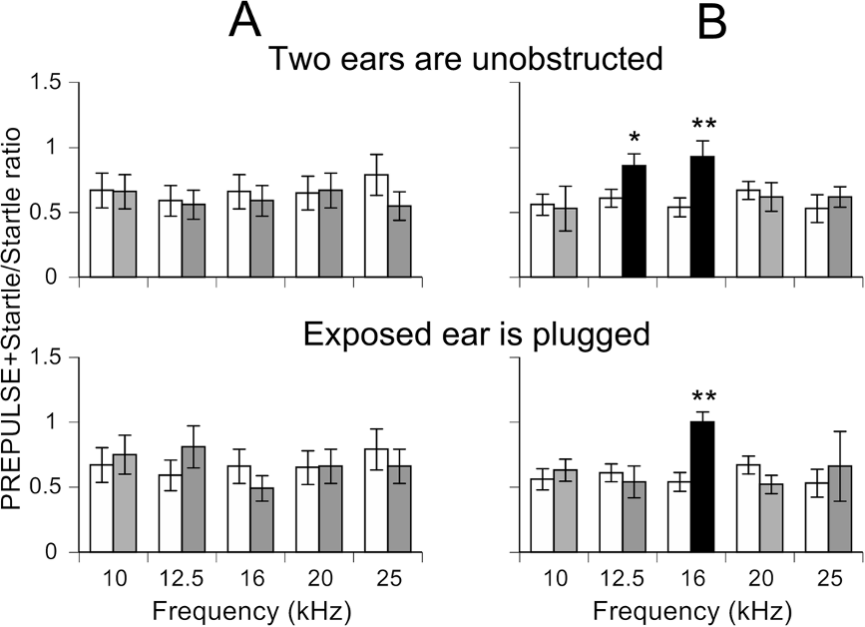
**Gap detection deficits in sound exposed mice were similar between the following conditions: When both ears are tested, or when only the protected ear was tested. (A)** Gap detection performance in a sound exposed mouse showing no gap detection deficits after exposure either when its two ears were unobstructed (top histogram) or when the exposed ear was plugged (bottom histogram). **(B)** A mouse showing similar frequency-dependent gap detection deficits when it was tested with the same paradigm as the mouse in *A*. Open bars represent mean ± SD of (G + S)/S ratios measured before sound exposure. Black and grey bars were and were not, respectively, significantly different from pre-sound exposure responses. *p < 0.05, **p < 0.001.

Each of the four mice tested demonstrated similar gap detection performance between testing conditions (Figure 7). A representative mouse in Figure 7A exhibited no significant changes in its gap detection performance after sound exposure when it was tested with two ears unobstructed. When the exposed ear was subsequently plugged, this mouse did not show detection deficits. The remaining three mice showed behavioral evidence of tinnitus. Likewise, their gap detection performance at the two testing conditions was similar. A representative mouse in Figure 7B showed robust gap detection deficits at 12.5 and 16 kHz when its two ears were unobstructed. These deficits remained evident at 16 kHz when the testing was performed with the exposed ear plugged.

## Discussion

Our results demonstrate that sound exposure triggers permanent changes in the mouse auditory system. The data presented provide evidence that mice can reliably be used in long-term GPIAS studies. ABR thresholds and pre-pulse inhibition ratios recovered to pre-exposure values after 84 days. In contrast, gap detection deficits and ASR magnitudes remained altered when tested through 360 days following acoustic exposure. In addition, our cochlear histology in combination with our plugged ear experiment demonstrated that the behavioral evidence of tinnitus observed in sound exposed animals cannot be easily explained by hearing loss caused by the exposure.

### Behavioral evidence of tinnitus remains in animals during 360 days of testing

Our previous study demonstrated that chronic tinnitus develops within 84 days following exposure to a loud narrow-band noise (Longenecker and Galazyuk 2011). This process is highly dynamic, with gap detection deficits evident over the entire frequency range used for testing (10 – 31.5 kHz) several days after exposure. Following a period of plasticity, the deficits were concentrated to a relatively narrow range at higher frequencies. Results of the present study show that at 180 and 360 days after exposure, gap detection deficits were shifted closer to the noise-exposure center frequency leading to deficits in a narrower range. Similar outcomes have been recently reported for a mixed C57BL/6 X 129 back-crossed mice strain (Turner et al. 2012). It was shown that chronic tinnitus started to emerge around 7 weeks after exposure and remained present until the 7 month period. This suggests that tinnitus development might be universal among mice, and if lifespan comparisons are made to humans, an important model of chronic tinnitus. One major inconsistency between these studies was that Turner et al. found diminished prepulse detection beginning at 4 months post exposure in both control and trauma mice. As the authors suggested this reduction can be explained by age related hearing loss, which has been well documented for C57BL/6 mice (Prosen et al. 2003), one of two stains that they crossed in their study. In the present study CBA/CaJ mice showed no sign of presbycusis. PPI values in these mice at 360 days post exposure were not significantly different from those recorded pre exposure. This was expected because CBA/CaJ mice preserve auditory functionality for more than one year of age (Idrizbegovic et al. 2001, Willott et al. 1994). Therefore, this mouse strain provides an opportunity to study age related effects on tinnitus development. For these reasons, CBA/CaJ mice could provide an excellent model for pharmaceutical agents aimed at treating chronic tinnitus.

### Cochlear histology fails to demonstrate loss of hair cells and spiral ganglia neurons

Results from our cochlear histology show the absence of noticeable damage to either hair cells or spiral ganglion neurons in sound exposed animals 360 days after sound exposure. The lack of hair cell loss following exposure is consistent with the results of several studies where the effect of sound exposure was determined in mice (Kujawa and Liberman 2009; Turner et al. 2012). In contrast, our study did not demonstrate significant loss of spiral ganglion neurons, which was reported previously (Kujawa and Liberman 2009). Key differences between our studies were the type of exposure and the age of the mice. We exposed mice under general anesthesia for one hour, whereas the latter study exposed awake mice for two hours. It is possible that younger mice in our study were more resistant to the exposure. However the lack of hair cells or ganglion neurons loss in our study does not exclude the possibility that some damage did occur, as we did not check the connectivity of ribbon synapses (Kujawa and Liberman 2009). This might explain why the magnitude of startle responses in our mice was reduced on the following day after exposure and remained suppressed during all year of testing.

### Does hearing loss contribute to gap detection deficits?

Over the last five years the GPIAS methodology for assessment of tinnitus in various laboratory animals (mice, rats, guinea pigs, and golden hamsters) has become increasingly utilized (Longenecker and Galazyuk 2011; Engineer et al. 2011; Coomber et al. 2014; Chen et al. 2013). This method is based on an assumption that the tinnitus is “filling in the gap” at the tinnitus frequency and subsequently reduce an animal’s ability to detect a gap, thus causing a reduction in gap-induced inhibition of the startle response (Turner et al. 2006). The literature on this method has provided controversial results. On one hand, many publications continue reporting that GPIAS is reliably detecting tinnitus-associated deficits in a subsample of animals that undergo various methods of tinnitus induction (Milbrandt et al. 2000; Turner et al. 2006; Yang et al. 2007; Dehmel et al. 2012). To the contrary, some studies show results that fail to support the “filling in the gap” assumption in animals (Hickox and Liberman 2014), and in human subjects with objective tinnitus (Campolo et al. 2013; Fournier and Hébert 2013). One possibility to explain gap detection deficits could be hearing loss resulting from acoustic trauma, the most common method of tinnitus induction. Monitoring a group of exposed mice for 360 days allowed us to address this important question. During the first 28 days following sound exposure all mice demonstrated signs of significant hearing deficits over a wide frequency range. This was evident from assessment of ABRs (Figure 4), gap detection (Figure 2), and PPI (Figure 3). The effect on PPI was most likely less severe than the effect on gap detection because prepulses are typically much more robust at inhibiting the startle than gaps in background noise. As time progressed, most signs of hearing loss dissipated except for decreased startle magnitude (Figure 5). Although this reduction was significant, it was high enough to be suppressed by a preceding gap or prepulse during our behavioral testing. Similarly, it has been shown that unexposed mice can have their startle magnitudes suppressed by continuous background narrow-band noise in a frequency dependent manner (Longenecker and Galazyuk 2012). However, if the suppressed startle magnitude stays above one standard deviation of the level of ambient animal movement, then the GPIAS methodology can be successfully used to assess gap detection performance. Reasons for why the startles are suppressed in exposed animals is difficult to ascertain, but could be explained by changes in peripheral and/or central parts of the auditory system, which cannot be detected by the tools used in this study. Until further research elucidates the causes for this suppression, unilateral sound exposure should be a reasonable choice for tinnitus induction.

Furthermore, our ear plug results argue against hearing loss as the major contributor to frequency-specific gap detection deficits in sound exposed animals. Our unilaterally exposed mice showed similar gap detection deficits when either both ears, or more importantly, just the unexposed ear, were tested. Since unexposed ears did not display any sign of ABR threshold increase after exposure, cochlear damage cannot be a reasonable explanation of the gap detection deficits. Although not completely definitive, this new finding further validates the gap detection methodology for tinnitus assessment. Alternatively, GPIAS might detect plastic changes along the auditory neuraxis caused by sound exposure, while not being capable of detecting tinnitus (Kaltenbach et al. 2005; Ma et al. 2006; Noreña and Eggermont 2003).

## Conclusions

Sound exposed mice retained gap detection deficits 360 days following exposure. The data presented provide evidence that mice can reliably be used in long-term GPIAS studies. Inner hair cells, outer hair cells, and spiral ganglion counts in the cochlea of control and sound exposed animals were not significantly different. The unilaterally plugged ear experiment helps demonstrate that the behavioral evidence of tinnitus observed in sound exposed animals cannot be easily explained by hearing loss caused by the exposure.

## Methods

### Subjects

Thirty male CBA/CaJ mice were used in this study. Twenty-two mice were from the original 84 day study (Longenecker & Galazyuk 2011) while eight mice were used for the addition of the unilateral plug study. Mice were obtained from Jackson Laboratories and were approximately 12 weeks old with a mean weight of 27.5 g at the beginning of testing. Mice were housed in pairs within a colony room with a 12-h light–dark cycle (8 A.M. to 8 P.M.) at 25°C. All procedures used in this study were approved by the Institutional Animal Care and Use Committee at Northeast Ohio Medical University.

### Acoustic trauma

Mice were anesthetized with an intraperitioneal injection of a ketamine/xylazine mixture (100/10 mg/kg). An additional injection (50% of the initial dose) was given intramuscularly 30 min after the initial injection. Eight mice were not exposed to serve as a control. Fourteen mice were exposed to a narrow-band noise centered at 16 kHz (4–22 kHz) unilaterally for 1 h. This noise was generated using a waveform generator (Wavetek model 395), amplified (Sherwood RX-4109) to 116 dB SPL, and played through a speaker (Fostex FT17H). The outputs of the loudspeaker were calibrated with a 0.25-in. microphone (Brüel and Kjaer 4135) and found to be ±4 dB between 10 and 60 kHz. A 2 mm O.D plastic tube was used to deliver sound from the speaker to the animal’s right ear. The left external ear canal was obstructed with a cotton plug. This manipulation typically reduces sound levels by at least 30 dB SPL to a level that does not induce tinnitus (Turner et al. 2006).

### Auditory brainstem response (ABR) testing

Mice were anesthetized with ketamine/xylazine as in the methods for acoustic trauma. ABR thresholds were obtained by presenting tone bursts at 10, 16, 20, 24, and 32 kHz at increasing sound intensities ranging from 10 to 80 dB SPL in 10 dB steps. Tones were 5 ms in duration, with 0.5 ms rise/fall time and delivered at the rate of 50/s. Auditory brainstem response (ABR) thresholds were obtained before and immediately after acoustic trauma. Sterile stainless-steel electrodes were placed subdermally, one behind the right pinna of the sound exposed ear and the other along the vertex. The unexposed ear was obstructed with a cotton plug. Evoked potentials were averaged over 300 repetitions. These potentials were amplified (Dagan 2400A preamplifier), filtered (100 – 3,000 Hz bandpass), digitized (HEKA Elektronik), and stored on a computer hard drive. Thresholds, the smallest sound amplitude that evoked a visible ABR, were determined by visually examining the averaged ABR waveforms in response to every sound frequency presented at different sound levels.

### Gap detection testing

The ability of mice to detect a gap of silence preceding the startle stimulus was determined using commercial hardware/software equipment from Kinder Scientific, Inc. High frequency anechoic foam was placed in each testing box to limit reverberations caused by the startling stimulus (Longenecker and Galazyuk 2012). Mice were placed in a plastic restrainer situated on a plate with a pressure sensor. Animal movement was detected by the sensor which measured its amplitude and stored data on the computer hard drive. Kinder Scientific software was used to generate a sequence of stimulus trials including a startle stimulus presented alone (STARTLE) and a startle stimulus paired with a gap (GAP + STARTLE) embedded into continuous background noise; the gap had a 20 ms duration and a 1 ms rise/fall time. Background for all trials was presented as a narrow-band (1/3 octave) noise centered at six different frequencies (10, 12.5, 16, 20, 25, and 31.5 kHz). Background noise level was constant (75 dB SPL) throughout the session. The startle stimulus was a 20 ms white noise presented at 110 dB SPL, with a 1 ms rise/fall time. The gap was 20 ms in duration and presented 100 ms before (onset to onset) the startle stimulus. Startle amplitude was measured as the peak-to-peak value (expressed in newtons (N)) during the 30-ms time window following startle stimulus onset.

For the gap detection test, parameters of our stimulus paradigm were set to levels which are typical for assuring a robust ∼ 30% reduction in startle response amplitude caused by a preceding gap of silence in an otherwise continuous background sound (Ison et al. 2002; Turner et al. 2006; Kraus et al. 2010; Longenecker and Galazyuk 2011).

The testing session started with an acclimation period lasting 3 min. Immediately afterwards, animals received 10 STARTLE-only trials in order to habituate their startle responses to a steady state level. For each of six background frequencies, we presented five STARTLE only trials and five GAP + STARTLE trials. The STARTLE and GAP + STARTLE trials were pseudo-randomized. The inter-trial intervals were also pseudo-randomized between 7 and 15 s. After we completed testing all six background frequencies, the entire session was repeated once more. Thus, during this testing for each background frequency, a total of 10 GAP + STARTLE trials and 10 STARTLE only trials were presented. All animals from the experimental group were tested before acoustic trauma, and then at several times points afterward: 1, 3–5, 7 days, weekly for 2 months, and at 84 days, 180 days, and 360 days post-exposure. The control group was tested at the same time points.

### Prepulse detection testing

The prepulse session contained two types of stimuli. First, a startle stimulus was presented in silence and had the same parameters as the startle during the gap detection session. The second stimulus type was the startle stimulus preceded by a prepulse. The prepulse stimuli were 20 ms in duration with a 1 ms rise/fall time and presented at the same six different narrow-band noise frequencies as in the gap detection session. For each frequency, mice were given five startle stimulus-alone trials, pseudo-randomized with five trials containing a prepulse. The amount of prepulse inhibition (PPI) was expressed as a ratio in the same way as the gap paradigm.

### Gap detection test with unilateral conductive hearing loss

Rationale for this study was to determine whether the sound exposed ear, which has potential hearing loss caused by exposure, contributes to our tinnitus assessment results. A separate group of 8 mice were sound exposed and 84 days post exposure they were assessed for the presence of tinnitus as described above. Based on these test results all mice were separated into two groups: tinnitus positive and tinnitus negative. All animals were then retested with the addition of an ear plug in the exposed ear installed under ketamine/xylazine anesthesia. Before testing, animals recovered from anesthesia in individual cages for two hours. This kind of a plug is known to attenuate sounds by 20–30 dB (Turner et al. 2006), and therefore significantly reduce the contribution of this ear to our test results. Results of these two assessments were compared for every animal from both groups.

### Data analysis

Startle responses showed some variability during the recording sessions: some animals sometimes exhibited an extremely strong startle response or did not startle at all. Therefore, the data in each session were statistically analyzed to remove outliers (Grubbs’ test for outliers). For each background frequency, a total of 10 GAP + STARTLE trials and 10 STARTLE only trials were presented. To calculate the GAP + STARTLE/STARTLE ratio we calculated mean for all startle values. They changed little within one session. Then we divided each of 10 GAP + STARTLE values for a given background frequency by the startle mean value. These 10 ratio values at a given frequency were used to calculate mean and SD values. These ratios are reported in Figures 1, 2, 3, and 7. A one-way analysis of variance (ANOVA) was used to test for differences within a subject. The criterion for the presence of behavioral evidence of tinnitus was a significant reduction in gap detection performance at one or several background frequencies compared to the pre-exposure values. During our data analysis, we found empirically that the 95% confidence interval is an optimal must-reach criterion to demonstrate changes in gap or prepulse detection performance induced by sound exposure.

### Cochlea histology

Following 360 days of tinnitus assessment mice were sacrificed for cochlear histology. Adult mice (n = 7) were anesthetized and transcardially perfused with 4% paraformaldehyde (PFA)/1x phosphate buffered saline (PBS). Temporal bones were dissected and post-fixed in the same solution overnight, then washed in 1x PBS and decalcified in 0.12 M EDTA for 5–7 days.

Cochleae were dissected and cut into base, middle, and apical regions (n = 3 mice). All cochlear segments were stained in whole mount with 1 μg/ml DAPI (4′,6′-diamidino-2-phenylindole; Sigma-Aldrich) for 3 mins in 1x PBS to visualize hair cells. Every segment was then photographed using a Leica DM5500B epifluorescence microscope and the total numbers of inner and outer hair cells counted on the photographs.

For SGN counts, temporal bones were dehydrated, embedded in paraffin, serially-sectioned in the longitudinal plane at 6 μm on a Leica microtome and mounted on Fisher Superfrost Plus slides (n = 4 mice). Consecutive sections through the entire spiral ganglion were stained with Mayer’s Hematoxylin/Eosin Y, and all SGNs with a clear nuclear membrane were counted on every fifth section (every 30 μm) at 400x magnification on a Leica DM5500B epifluorescence microscope. The 16-20 kHz region was identified according to published criteria (Kujawa and Liberman 2009). To correct for overcounting of SGNs, digital photographs of all regions of the spiral ganglion were taken in a single mid-modiolar section using a Carl Zeiss Axiocam, and nuclear diameters (160-250/cochlea) were measured using ImageJ software (NIH). The Hendry method (Hendry 1976) was used to correct for overrepresentation of nuclei in multiple sections.

Average hair cell and SGN numbers were calculated and compared using two-tailed t-tests (Microsoft Excel). All counts were conducted blinded to treatment status and are reported as average ± SD.

## Abbreviations

GPIAS: Gap-induced pre-pulse suppression of the acoustic startle
PPI: Prepulse inhibition
ASR: Acoustic startle reflex
OHC: Outer hair cell
IHC: Inner hair cell
SGN: Spiral ganglion neuron
(G + S)/S: (Gap + startle)/startle
PFA: Paraformaldehyde
PBS: Phosphate buffered saline
ABR: Auditory brainstem response
DAPI: 4',6'-diamidino-2-phenylindole.
